# Gut microbiome characteristics of comorbid generalized anxiety disorder and functional gastrointestinal disease: Correlation with alexithymia and personality traits

**DOI:** 10.3389/fpsyt.2022.946808

**Published:** 2022-08-16

**Authors:** Xunyi Guo, Feng Lin, Fengjiao Yang, Jing Chen, Weixiong Cai, Tao Zou

**Affiliations:** ^1^Department of Psychiatry, The Affiliated Hospital of Guizhou Medical University, Guiyang, China; ^2^Department of Clinical Medicine, Guizhou Medical University, Guiyang, China; ^3^Department of Psychiatry, The Second Affiliated Hospital of Xinxiang Medical University, Xinxiang, China; ^4^Shanghai Key Lab of Forensic Medicine, Key Lab of Forensic Science, Ministry of Justice, Shanghai Forensic Service Platform, Academy of Forensic Science, Shanghai, China

**Keywords:** gut microbiome, functional gastrointestinal disorders (FGIDs), general anxiety disorders (GAD), 16S rRNA, comorbidity

## Abstract

**Objective:**

The aim of this study was to investigate the characteristics of intestinal flora in patients with functional gastroenteropathy and generalized anxiety disorder (GAD) and the relationship between intestinal flora and psychological factors.

**Materials and methods:**

From March 2020 to December 2020, a total of 35 patients with functional gastroenteropathy and generalized anxiety disorder, 30 healthy controls, 16 patients with functional gastroenteropathy, and 44 patients with generalized anxiety disorder were selected from the Affiliated Hospital of Guizhou Medical University. Fecal samples were collected from each group, and the related psychophysiological factors scales (Hamilton Anxiety Scale, Hamilton Depression Scale, Neurotic Personality Questionnaire, concept of illness questionnaire, Toronto Alexithymia Scale, Severity of Physical Symptoms Scale, and Cognitive Emotion Regulation Questionnaire) were improved. 16S rRNA high-pass sequencing was used to determine the correlation between intestinal flora changes and functional gastroenteropathy with generalized anxiety disorder. Then, the scale and gut microbiota results were analyzed for correlation to determine the correlation between personality traits and gut microbiota.

**Results:**

We found similar intestinal microbiota in patients with functional gastroenterology, generalized anxiety disorder, and functional gastroenteropathy with generalized anxiety disorder. But the relative abundance of Clostridium was significantly increased in patients with functional gastrointestinal disease (FGID) and generalized anxiety. The relative abundance of *Haemophilus influenzae* was significantly increased in patients with functional gastrointestinal disease without a generalized anxiety disorder. The intestinal microecological composition was significantly correlated with personality traits.

**Conclusion:**

Functional gastrointestinal disease comorbidity GAD may be related to an increase in the relative abundance of *Fusobacterium*. FGID non-comorbidity GAD may be related to the increased relative abundance of *Hemophilus*. The increased relative abundance of *Fusobacterium* and *Megamonas* is associated with personality traits such as difficulty describing feelings and difficulty identifying feelings, neuroticism, and negative cognition of disease.

## Introduction

Generalized anxiety disorder (GAD) is characterized by persistent and significant tension and restlessness, accompanied by autonomic nervous function excitation and hypervigilance that persists for most of at least 6 months. The prevalence of generalized anxiety disorder was 13.6%, with a significantly higher prevalence in women than in men (1). It is also prolonged, prone to recurrence, and has a significant impact on the physiological and psychological functioning of the individual.

Functional gastrointestinal disease (FGID) is a clinically common gastrointestinal dysfunctional disorder that affects approximately one-third of the world’s population (2). FGIDs are characterized by a variety of chronic, recurrent clusters of gastrointestinal symptoms, and the corresponding organic lesions cannot be detected by various medical examinations. FGID mainly includes functional dyspepsia (FD), irritable bowel syndrome (IBS), functional constipation (FC), functional bloating (FB), and so on. FGID is characterized by chronic or recurrent gastrointestinal symptoms, and these symptoms cannot be explained by known pathophysiological mechanisms (3). The pathogenesis of FGID is still unclear and is thought to be due to a combination of multiple factors. Psychosocial factors are thought to play a key role in its pathogenesis (4, 5).

Studies have shown that mood disorders can predict the development of functional gastrointestinal disorders (FGIDs), which in turn predict the development of mood disorders (6). The prevalence of comorbid anxiety disorders in patients with FGID is estimated to be more than 40% (7, 8). A recent meta-analysis similarly showed that the prevalence of anxiety symptoms in patients with FGIDs was 39.1% (9). Studies have shown a significant positive correlation between the degree of anxiety and depression and the grading of gastrointestinal symptoms in patients with FD, IBS, and FC (10). The prevalence of anxiety and depression increases progressively with the number of coexisting FGID disorders and the frequency and severity of gastrointestinal symptoms (11). Cognitive behavioral therapy and mood-improving medications may help alleviate gastrointestinal symptoms in patients with FGID (12). Anxiety was shown to be an independent predictor of first-episode FGIDs in a 12-year prospective study, and patients diagnosed with FGIDs also had higher anxiety during follow-up (13). These studies suggest that anxiety disorders and FGIDs often have comorbidities, but the pathophysiological mechanism of comorbidities is still unclear and needs to be further explored.

With the concept of the microbial-gut-brain axis, the role of the gut microbiome in gastrointestinal and psychiatric diseases has received increasing attention from scholars. The gut microbiome regulates the brain function and behavior of the host through the microbial-gut-brain axis, and the changes in the gut microbiome also affect the development and function of the central nervous system (CNS) through the microbial-gut-brain axis (14). In animal experiments, feces from chronically unpredictable mildly stressed mice were transplanted to control mice, and the recipient mice showed anxiety-like and depression-like behaviors similar to those of the donor mice (15). Studies have shown that the gut flora of patients with a generalized anxiety disorder has reduced alpha diversity compared to healthy populations and that there is a positive correlation between anthropoid genera and *Escherichia coli*-Shigella and anxiety severity (16). In patients with FGID, studies have shown that dysbiosis of small intestinal flora may be the basis of symptoms of functional gastroenteropathy (17). In addition, diarrhea, IBS, and depression patients have similar intestinal flora (18). Transplanting feces from a healthy person to FGID patients with anxiety or depression improves mood and gastrointestinal symptoms (19). These findings suggest that the gut microbiome may play a key role in the physiological mechanism of comorbid GAD and FGID. However, few reports have been reported on the differences in gut microbiomes in FGID, GAD, and their comorbidities. In addition, personality plays a key role in mental health, and studies have shown genetic and biological links between mental health and personality (20). In addition, there are significant differences in pain duration, severity, and dysfunction of IBS disease in patients with different personalities (21). People with negative perceptions of the disease and a lack of expression of their own emotions are often more likely to develop functional gastroenteropathy disorders (8). Understanding the role of personality on gut microbiota can help us understand the role of mental health on gut microbiota.

This study was conducted to investigate the relationship between the diversity of intestinal flora and psychological factors in patients with functional gastrointestinal disease and generalized anxiety disorder, based on the intestinal flora-gut-brain axis, and to find the key bacteria associated between them. We further analyzed the correlation between intestinal flora and psycho-behavior as well as personality in order to provide a theoretical basis and a new target for intervention in the treatment and prognosis evaluation of functional gastrointestinal disease with generalized anxiety disorder.

## Materials and methods

### Participants

Written consent has been obtained from all patients before specimen collection. The study was reviewed and approved by the Medical Ethics Committee of the Affiliated Hospital of Guizhou Medical University and was in accordance with the Declaration of Helsinki. Patients with FGID and GAD in the psychiatric and gastroenterology inpatient department of the Affiliated Hospital of Guizhou Medical University were selected. The inclusion criteria were as follows: ① no history of other psychiatric disorders and psychoactive substance abuse; ② no serious organic disease; ③ ages 18–65. The exclusion criteria were as follows: ① previous history of psychoactive substance abuse; ② combined with serious organic diseases; and ③ pregnant or lactating women. Subsequently, according to the diagnostic criteria of functional gastroenteropathy (Rome IV), the diagnostic criteria of a generalized anxiety disorder (ICD-11), and the Hamilton anxiety scale score, they were divided into generalized anxiety disorder group (ADD), functional gastroenteropathy group (FGID), and functional gastroenteropathy with generalized anxiety disorder group (FAD). The inclusion criteria of the control group (CG) were ① family members living in the same area as the experimental group; ② age 18–65 years; ③ no previous history of psychiatric and gastrointestinal diseases; ④ laboratory routine examination (blood, urine, stool routine, liver function, and blood lipid) was normal. The exclusion criteria were the same as those for the experimental group.

### Sample collection

After completing the questionnaire test, fresh feces from patients and volunteers were collected, and feces from 30 healthy controls, 16 patients with functional gastroenteropathy, 44 patients with generalized anxiety disorder, and 35 patients with comorbid GAD and FGID were collected in 50 ml sterile disposable tubules. The feces were numbered, packed in ice boxes, and transferred to the laboratory refrigerator at -80°C for standby.

### Screening tool

(1) Hamilton Anxiety Scale (HAMA-14) (22): It was developed by Hamilton in 1959, and it is the most common scale used in the clinical assessment of anxiety. The reliability coefficient of this scale was 0.9, and the validity coefficient of the scale was 0.36, respectively. It included 14 items on a 5-point scale from 0 to 4: (0) asymptomatic, (1) mild, (2) moderate, (3) severe, and (4) extremely severe.

(2) Five Factors Inventory-Neuroticism Subscale (FFI-N) (23): The scale adopts the neuroticism dimension of the Big Five personality; it has 12 items in the questionnaire and is rated on a 5-point scale from 1 to 5: (0) completely disagree, (1) disagree, (2) have no opinion, (3) agree, and (4) completely agree. The scale is widely used in China, and higher total scores indicate the more obvious neuroticism characteristics. The internal consistency coefficient of the FFI-N in this study was Cronbach’s alpha = 0.921.

(3) The Chinese version of Illness Perception Questionnaire Revised (IPQ-R): This scale was developed by Weinman et al. (24). In 2002, it was revised and formed IPQ-R through Moss-Morris et al. This study adopts the Chinese version translated by Xiong et al. (25). The questionnaire consisted of three subscales, and the second subscale was used in this study. There were 38 items in the questionnaire, including seven dimensions of the course of disease (acute and chronic), consequences, personal control, treatment control, disease consistency, disease periodicity, and emotional statement. It is rated on a 5-point scale from 1 to 5: (0) totally disagree, (1) disagree, (2) have no opinion, (3) agree, and (4) strongly agree. Among them, the course of disease (acute and chronic), consequences, disease periodicity, and emotional statements belong to negative sexiness knowledge. The higher the score is, the greater the negative sexiness knowledge is. Personal control, treatment control, and disease consistency are positive perceptions, and the higher the score, the stronger the positive perceptions.

(4) The Twenty-Item Toronto Alexithymia Scale (TAS-20): This scale was developed by Bagby et al. (26). This study adopted the revised version by Yi et al. (27), which includes 20 items in total. It contains three dimensions, namely, difficulty describing feelings, difficulty identifying the feeling, and externally oriented thinking. The difficulty describing feeling subscale is used to measure difficulty describing emotions (five items); the difficulty identifying feeling subscale is used to measure difficulty identifying emotions (seven items); and the externally oriented thinking subscale is used to measure the tendency of individuals to focus their attention externally (eight items). The scale was rated on a 5-point scale from 1 to 5: (0) completely disagree, (1) disagree, (2) have no opinion, (3) agree, and (4) completely agree. The retest reliability of the scale was 0.87.

(5) Patient Health Questionnaire-15 (PHQ-15) (28): This scale consists of the 15 most common somatic symptoms and is used to assess the severity of somatic symptoms in the last month, using a 3-grade scoring method of 0–2, with a total score ranging from 0 to 30 points, in which ∼4 points represented no somatic symptoms, 5–9 points represented mild somatic symptoms, 10–14 points represented moderate somatic symptoms, and ≥15 points represented severe somatic symptoms. The internal consistency reliability coefficient was 0.73, and the retest reliability coefficient was 0.75.

(6) Cognitive Emotion Regulation Questionnaire-Chinese Version, CERQ (29): This scale is used to assess the cognitive strategies used by individuals in coping with negative events. The scale contains 36 entries, including acceptance, positive attention, plan of attention, positive reappraisal, rational analysis, self-blame, self-reflection, catastrophizing, and blaming others in nine dimensions using a 5-level scoring method. The higher the total score on the scale is, the lower the individual’s cognitive emotion regulation level is. The Cronbach’s α coefficient of the CERQ-C scale was 0.8 L, and the retest reliability of the full scale was 0.56.

### Sequencing of 16srDNA amplicons

DNA was extracted from stool samples using the PowerSoil R^®^DNA isolation kit (Qiagen, Hilden, Germany) according to the manufacturer’s recommended protocol. Primers 515F (5’-ACTCCT ACGGGAGgCAGCAGG-3’) and 806R (5’-GGactachVGGGTWTCtaat-3’) were used for PCR amplification of the v3-V4 variable region of bacterial 16S rRNA gene, and the PCR products were quality controlled with a 2% concentration agarose gel; NovaSeq PE250 (Illumina) was used for up-sequencing.

### Bioinformatics processing

The original sequences obtained from Illumina NovaSeq were assembled, screened, and chimeras constructed according to the Qiime (V.1.9.1) quality control process. OTUs clustering of valid data with 97% consistency (Identity) was performed using the Uparse software (version 7.0.1001). The SILVA database (V123) was used as a reference for species annotation of OUT sequences with species using classifiers. Shannon, Simpson, Chao1, ACE, Goods_Coverage, PD_whole_tree, and other analysis indexes were used to evaluate α diversity. Weighted Unifrac and unweighted Unifrac were used to calculate β diversity. Weighted Unifrac distance and Unweighted Unifrac distance were used for PCoA analysis. To overcome the shortcomings of linear models (including PCA and PCoA), NMDS analysis was conducted based on Bray-Curtis distance to explore the differences in community structure among different samples or groups.

### Statistical methods

The *SPSS 19.0* software was used for statistical analysis of the study data. Quantitative data were expressed as −*x* ± *s*, grade data were expressed as frequency (*%*), and an independent *t*-test and one-way ANOVA were used for comparison between groups. The differences in intestinal flora between groups were analyzed by alpha diversity index and LDA Effect Size (LEfSe), and the scatter plot was based on a non-metric multidimensional scale (NMDS). Spearman correlation was used to analyze the correlation between intestinal microflora and psychological factors. *P* < *0.05* indicated that the difference was statistically significant.

## Results

### Demographic characteristics

A total of 125 subjects were recruited for this study, including 30 healthy subjects (CG), 16 in functional gastroenteropathy (FGID), 44 in generalized anxiety disorder group (ADD), and 35 in functional gastroenteropathy with a generalized anxiety disorder (FAD). The proportion of women in the ADD group (77%) was higher than in other groups. The proportion of Han nationality in the FGID group (75%) was higher than in the other groups. The proportion of smoking in the functional gastroenteropathy with generalized anxiety disorder group (23%) was higher than that in other groups. The proportion of drinking and city in the CG group (37 and 57%) was higher than that in other groups, but there was no statistical difference (Table 1).

**TABLE 1:** Clinical parameters and demographic information of subjects.

	CG (*n* = 30)	FGIDS (*n* = 16)	ADD (*n* = 44)	FAD (*n* = 35)	合计 (*n* = 125)	*F*	*P*
Age	40.2 ± 6.4	37.6 ± 11.8	35.3 ± 9.5	34.4 ± 10.2	36.5 ± 9.6	2.435	0.068
Sex (female%)	73	50	77	54	67	2.468	0.065
BMI	23.1 ± 2.3	21.4 ± 3.4	21.4 ± 3.0	21.4 ± 3.5	21.8 ± 3.1	2.403	0.068
Race (%)						0.478	0.698
Han	60	75	66	71	67		
Others	40	25	34	29	33		
Smoking (%)	13	13	14	23	16	0.560	0.642
Drinking (%)	37	18	27	31	30	0.587	0.625
Residence (%)						0.654	0.582
Urban	57	38	43	49	47		
Rural	43	62	57	51	53		
Family history (%)	0	0	9	11	6	1.735	0.163
Formerly medical history (%)	3	19	14	11	11	1.008	0.392

### Characteristics of intestinal flora

**FIGURE 1:**
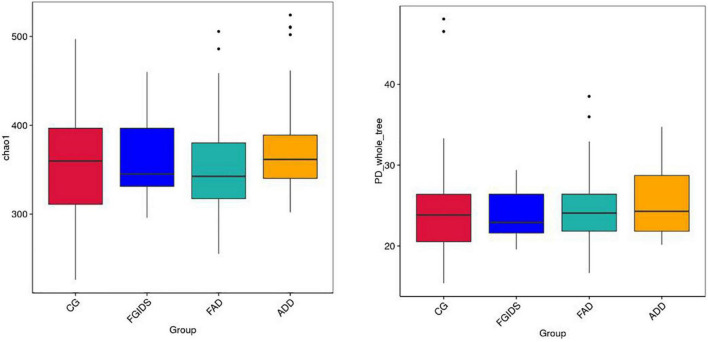
Analysis chart of α diversity of intestinal flora among different groups.

**FIGURE 2:**
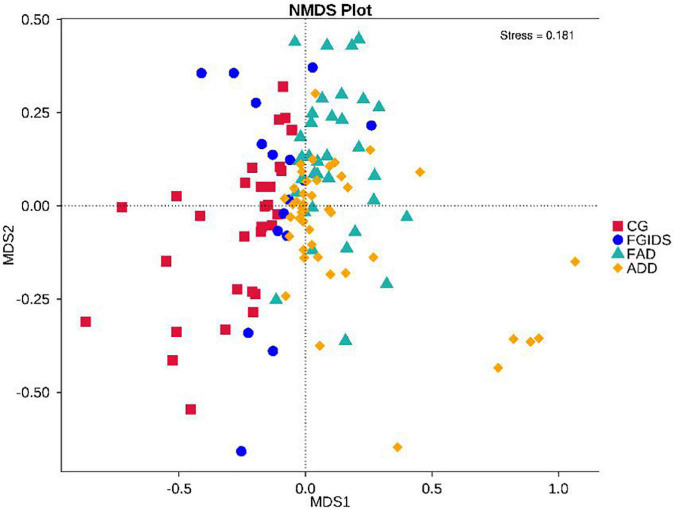
Analysis chart of β diversity of intestinal flora among different groups.

To explore the intestinal microbiota associated with GAD and FGID, the α diversity index of the total sample was analyzed. According to the chao1 index and PD whole_tree index analysis, there was no significant difference in species richness among groups [all *P-*values > 0.05 (Figure 1)]. NMDS analysis of β diversity based on Bray-Curtis distance revealed differences in the composition of intestinal flora between the four groups (Figure 2).

**FIGURE 3:**
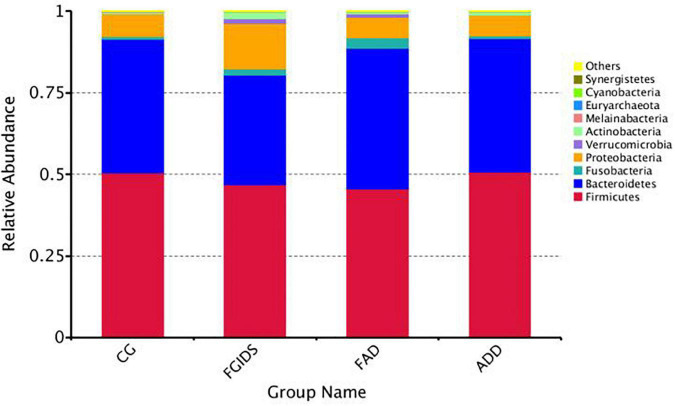
Phylum level species stack map.

**FIGURE 4:**
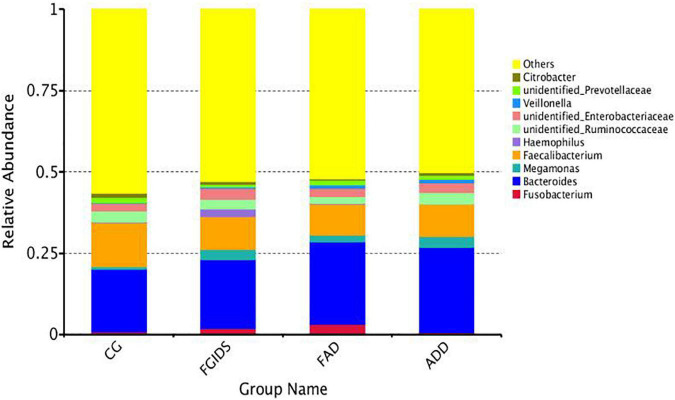
Stack diagram of horizontal species distribution of genus.

At the phylum level, the dominant bacteria attached to CG, FGID, FAD, and ADD groups were basically the same. Firmicutes, Bacteroidetes, Fusobacteria, and Proteobacteria occupy almost 98% of the intestinal flora. In the CG group, the proportion was 50.48, 40.79, 0.8, and 0.69%; in the ADD group, the proportion was 50.69, 41.04, 0.7, and 6.3%. In the FGID group, the proportion was 46.97, 33.57, 1.9, and 13.77%. In the FAD group, the proportion was 45.57, 43.16, 3.13, and 6.3%. We found that the abundance of Fusobacteria in the CG and ADD groups was relatively lower than in the FGID and FAD groups (0.8% CG and 0.7% ADD, 46.97% FGID, and 45.57% FAD) (Figure 3). At the genus level, *Fusobacterium*, *Bacteroides*, *Megamonas*, *Faecalibacterium*, *Haemophilus*, and Unidentified Ruminococcaceae were the dominant bacteria. In the CG group, the proportion was 0.8, 19.28, 0.8, 13.61, 0.2, and 3.3%; in the ADD group, the proportion was 0.7, 26.19, 3.2, 10, 0.8, and 3.6%; in the FGID group, the proportion was 1.9, 21.20, 3.1, 10.1, 2.42, and 3%. In the CG group, the proportion was 3.13, 25.38, 2.1, 9.54, 0.1, and 2.3%. We found that the abundance of Fusobacterium in the CG and ADD groups was significantly lower than that in the FGID and FAD groups (0.8% CG, 0.7% ADD, 1.9% FGID, and 3.13% FAD), and the abundance of Fusobacterium in the FGID group was also significantly lower than in the FAD group (Figure 4).

**FIGURE 5:**
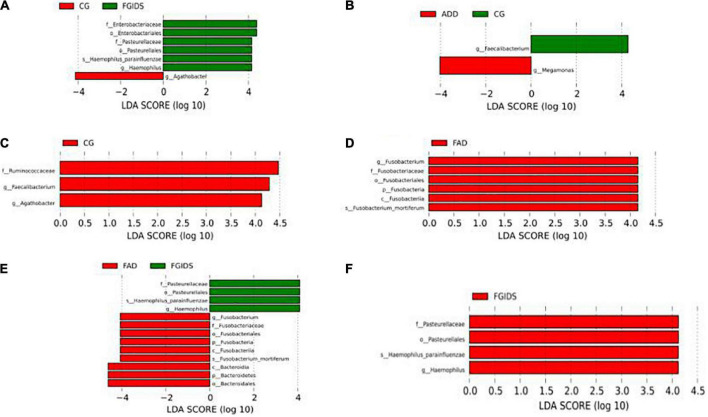
Analysis of significant difference flora among different group. **(A)** CG-FGIDS. **(B)** ADD-CG. **(C)** FAD-CG. **(D)** ADD-FAD. **(E)** ADD-FGIDS. **(F)** FAD-FGIDS.

The LEfSe method further confirmed the difference in intestinal flora structure among groups. Ruminococcaceae, *Faecelibacterium*, and *Agathobacter* in the FAD group decreased significantly compared with the CG group, while Fusobacterium in the FAD group increased significantly compared with the ADD group. Fusobacterium and Bacteroid in the FAD group were significantly increased compared with the FGID group, while *Pasteurella*, *Hemophilus*, Parainfluenzae, and *Hemophilus* in the FAD group were significantly decreased compared with the FGID group. *Pasteurella*, *Hemophilus*, Parainfluenzae, and *Hemophilus* were significantly increased in the FGID group compared with the ADD group. *Enterobacterium*, *Pasteurella*, *Haemophilus*, Parainfluenzae, and *Haemophilus* in the FGID group were significantly increased compared with the CG group, while *Aathobacter* was significantly decreased compared with the CG group. Faecalibacterium in the ADD group decreased significantly compared with the CG group, while Megamonas increased significantly compared with the CG group (Figure 5).

### Correlation analysis of psychogenic factors and intestinal microflora

The study showed that the scores of TAS, PHQ, IPQ (negative), FFIN, and HAMA in the FAD group were significantly higher than those in the FGID group. The PHQ score in the FAD group was significantly higher than that in the ADD group. The scores of TAS, PHQ, IPQ (negative), FFIN, and HAMA in the ADD group were significantly higher than those in the FGID group (Table 2). A Spearman correlation analysis was used to analyze the relationship between scale scores of TAS and the abundance of bacteria. The results showed that TAS scores were significantly positively correlated with *Fusobacterium*, *Megamonas*, and *Veillonella* (*P* < 0.01). PHQ scores were positively correlated with *Fusobacterium* and *Veillonella* (*P* < 0.01) and were significantly negatively correlated with *Faecalibacterium* and Ruminococcaceae (*P* < 0.01). FFI-N scores were significantly positively correlated with *Fusobacterium*, *Megamonas*, *Veillonella*, and Enterobacteriaceae (*P* < 0.01) and were negatively correlated with *Faecalibacterium* (*P* < 0.01). HAMA scores were significantly positively correlated with *Fusobacterium*, *Veillonella*, and *Megamonas* (*P* < 0.01), and *Bacteroides* and Enterobacteriaceae (*P* < 0.05). HAMA scores were significantly negatively correlated with *Faecalibacterium* (*P* < 0.01) and Ruminococcaceae (*P* < 0.05). The CERQ scores were significantly positively correlated with *Megamonas* (*P* < 0.01). The IPQ-R scores were significantly positively correlated with *Fusobacterium*, *Megamonas*, and *Veillonella* (*P* < 0.01) were significantly negatively correlated with *Faecalibacterium* and Ruminococcaceae (*P* < 0.01) (Table 3).

**TABLE 2:** Comparison of scale scores among groups (*−x* ± *s*).

项目	CG组 (*n* = 30)	FGIDS组 (*n* = 16)	ADD组 (*n* = 44)	FAD组 (*n* = 35)	*F*
TAS	27.53 ± 2.67	52.25 ± 8.01^a^	57.75 ± 7.45^ab^	61.14 ± 10.54^ab^	121.79
PHQ	2.33 ± 0.55	9.19 ± 4.43^a^	13.59 ± 4.77^ab^	17.11 ± 4.81^abc^	76.04
IPQ (负性)	22.50 ± 0.68	65.13 ± 6.54^a^	71.52 ± 8.96^ab^	72.86 ± 8.54^ab^	331.76
IPQ (正性)	16.17 ± 0.38	50.06 ± 4.17^a^	51.18 ± 5.13^a^	50.09 ± 4.63^a^	512.35
FFIN	12.10 ± 0.31	32.44 ± 5.11^a^	36.77 ± 4.62^ab^	37.57 ± 4.77^ab^	264.74
HAMA	4.93 ± 0.87	17.56 ± 2.06^a^	38.66 ± 9.28^ab^	39.80 ± 10.13^ab^	152.738
CERQ	37.33 ± 2.36	106.81 ± 16.56^a^	109.52 ± 13.11^a^	109.71 ± 17.66^a^	212.42

Compared to the CG group, ^a^*P* < 0.05; Compared to the FGID group, ^b^*P* < 0.05; Compared to the ADD group, ^c^*P* < 0.05.

**TABLE 3:** Correlation analysis of psychosocial factors and intestinal flora (The Rs values).

Type	TAS	PHQ	FFIN	HAMA	CERQ	IPQ	IPQ (正性)	IPQ (负性)
*Fusobacterium*	0.345**	0.336**	0.304**	0.314**	0.209*	0.340**	0.218*	0.299**
*Bacteroides*	0.185*	0.161	0.180*	0.187*	0.068	0.114	0.112	0.046
*Megamonas*	0.284**	0.175	0.326**	0.312**	0.233**	0.324**	0.259**	0.381**
*Faecalibacterium*	-0.148	-0.236**	-0.214*	-0.251**	-0.140	-0.259**	-0.231**	0.196*
*Haemophilus*	-0.032	-0.070	-0.019	-0.161	-0.055	0.019	-0.062	0.058
Unidentified_Ruminococcaceae	-0.184*	-0.233**	-0.140	-0.228*	-0.013	-0.226*	-0.101	-0.159
Unidentified_Enterobacteriaceae	0.058	0.208*	0.301**	0.185*	0.156	0.202*	0.182*	0.188*
*Veillonella*	0.243**	0.242**	0.279**	0.251**	0.163	0.315**	0.298**	0.316**

**P < 0.01; *P < 0.05.

**FIGURE 6:**
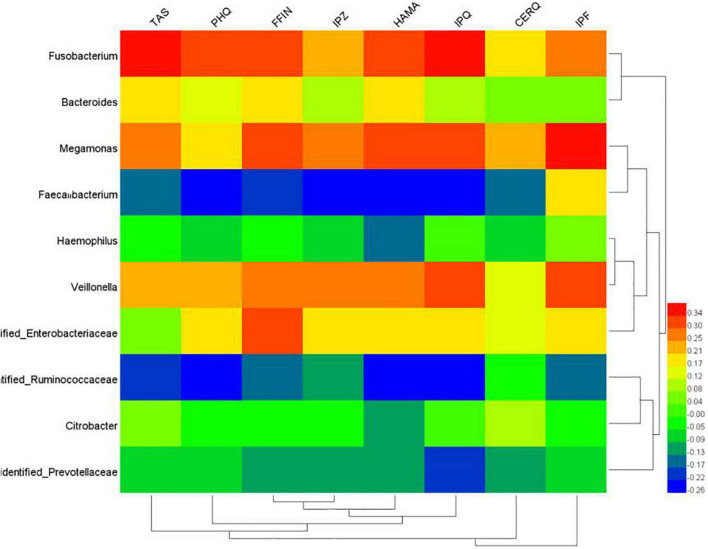
Heat map of correlation analysis between psychosocial factors and intestinal flora.

The heat map shows that *Fusobacterium*>*Megamonas*>*Veillonella* were closely related to anxiety. *Fusobacterium*>*Veillonella* was closely correlated with somatic symptoms. *Megamonas* was closely related to the negative perception of the disease, and *Veillonella* was closely related to the positive knowledge of the disease (Figure 6).

## Discussion

Functional gastrointestinal disease is a common and multifaceted disease of the digestive system with symptoms as the main diagnostic basis and is influenced by multiple factors. The results of current studies have found that psychological factors play a key role in the pathogenesis of FGID. Currently, psychological factors have been found to play a key role in the pathogenesis of FGID, but the changes in intestinal flora in patients with FGID and GAD comorbidities have been less studied.

The results of this study showed that the abundance of *Fusobacterium* in the FAD and FGID groups was relatively higher than that in the ADD and CG groups. Furthermore, LEfSe analysis revealed that the relative abundance of *Fusobacterium* in the ADD and FAD groups was significantly lower in the FGID group. *Fusobacterium* is considered as a pathogen because of its invasive and inflammatory nature, its ability to enter the bloodstream and cause intestinal barrier dysfunction, and its role in immune activation of GAD. To the best of our knowledge, this is the first report of *Clostridium perfringens* alterations in patients with functional gastrointestinal disease comorbidities and generalized anxiety. However, it was found that the relative abundance of Fusobacterium and Bacteroid in patients with GAD was higher than that in healthy people (30). Compared with healthy people, the relative abundance of *Fusobacterium* in the intestinal flora of patients with IBS increased (31). Also, this study showed that *Fusobacterium* and *Veillonella* were significantly and positively correlated with the severity of somatic symptoms (*P* < 0.01), and the correlation coefficients were (*R* = 0.336, 0.242), respectively. It indicates that the higher the percentage in the intestinal tract, the more severe the patient’s physical symptoms, aggravating the patient’s disease. *Fusobacterium*, *Megamonas*, and *Veillonella* were significantly positively correlated with anxiety scores (*P* < 0.01). The correlation coefficients were 0.314, 0.312, and 0.251, respectively. Therefore, we suggested that the increase in the relative abundance of Fusobacterium in intestinal flora might be related to comorbid GAD and FGID.

The results of LEfSe analysis in this study showed that the relative abundance of Hemophilus in the CG, ADD, and FAD groups was significantly lower in the FGID group. Previous studies have shown that the relative abundance of Hemophilus is elevated in patients with functional gastroenterology compared with healthy people (32). Therefore, we speculated that the elevated relative abundance of Hemophilus in the intestinal flora might be related to the pathogenesis of FGID without GAD. Hemophilus is considered to be one of the main pathogens responsible for chronic inflammation. Somatic inflammatory reaction is believed to be the cause of FGID (33), but no relevant study has reported the correlation between the increased relative abundance of Hemophilus and FGID without GAD, which is worthy of further exploration.

Studies have shown that psychosocial factors are associated with disease awareness, difficulty describing feelings, symptom severity, and outcome in patients with FGIDs (8). Patients with different personalities have significant differences in the duration, severity, and functional impairment of their IBS disorders (21). Neuroticism and covert aggression are the most likely markers of FGID susceptibility (34). This study further revealed that the scores of TAS, PHQ, IPQ (negative), FFIN, and HAMA in the FAD group were significantly higher than those in the FGID group through correlation analysis of different groups and scales. The results showed that the severity of physical symptoms and the negative cognitive characteristics of the disease were prominent, the level of anxiety and depression was high, the neuroticism was more obvious, and the difficulty describing feelings and difficulty identifying feelings were more likely. The results of a recent meta-analysis suggest that the mental health of inflammatory bowel disease patients is associated with inflammatory bowel disease outcomes (35). Functional gastroenterology patients with alexithymia tend to have more severe somatic symptoms (36). Enhanced perception of visceral stimulation is considered to be one of the key features of IBS. Alexithymia may enhance visceral hypersensitivity in IBS (3). People with severe alexithymia tend to amplify somatosensation (37). These findings support a significant association between FGID and psychological factors, but the role of gut microbiota remains to be explored. The Spearman correlation analysis showed that TAS scores were significantly positively correlated with *Fusobacterium*, *Megamonas*, and *Veillonella* (*P* < 0.01). The FFI-N score was significantly positively correlated with *Fusobacterium* and *Megamonas* (*P* < 0.01). The CERQ score was positively correlated with *Megamonas* (*P* < 0.01). The IPQ-R scores were significantly positively correlated with *Fusobacterium*, *Megamonas*, and *Veillonella* (*P* < 0.01). The composition of intestinal microecology is correlated with personality traits (38). People with high neuroticism had significantly lower gut microbiome β diversity than those with low neuroticism (39). Therefore, we believe that the increased relative abundance of *Fusobacterium* and *Megamonas* is associated with personality traits such as difficulty describing feelings and difficulty identifying feelings, neuroticism, and negative cognition of disease and that the comorbidity of GAD and FGID is more likely to occur.

The shortcomings of this study are the small sample size and the single detection method (only 16S high-throughput sequencing). To compensate for the above deficiencies, on the one hand, this study will expand the sample size, further explore whether the intestinal flora is specific for psychological effects, establish FGID with GAD and GAD population cohort, and further group FGID with GAD with or without antipsychotic drugs, in order to analyze the effects of antipsychotic drugs on the intestinal flora. On the other hand, metabolomic analysis was performed on the samples to screen for significantly different metabolites as potential biomarkers for FGID and GAD and to establish a link between intestinal peptide-mental disorders. Meanwhile, the role of probiotics in patients with comorbid GAD and FGID will also be explored, so as to elucidate the mechanism of the “intestinal microbiome-gut-brain” axis.

## Conclusion

Functional gastrointestinal disease comorbidity GAD may be associated with an elevated relative abundance of Fusobacterium. FGID non-comorbidity GAD may be related to the increased relative abundance of Hemophilus. The increased relative abundance of *Fusobacterium* and *Megamonas* is associated with personality traits such as affective difficulty describing feelings and difficulty identifying feelings, neuroticism, and negative cognition of disease.

## Data availability statement

The data presented in the study are deposited in the SAR repository, accession number PRJNA 863752.

## Ethics statement

The studies involving human participants were reviewed and approved by the Medical Ethics Committee of the Affiliated Hospital of Guizhou Medical University. The patients/participants provided their written informed consent to participate in this study.

## Author contributions

XG drafted the manuscript for submission. XG and FL were responsible for data collection, the study’s topic and scope, and research of evidence as well as analysis and interpretation of data. JC and FY were involved in data collection, coding of data, and provided intellectual input into the manuscript. TZ and WC supervised the study and provided intellectual input into the manuscript. All authors read and approved the final manuscript.
